# Facilitating English Grammar Learning by a Personalized Mobile-Assisted System With a Self-Regulated Learning Mechanism

**DOI:** 10.3389/fpsyg.2021.624430

**Published:** 2021-10-07

**Authors:** Xiao Wang, Jing Chen, Tingting Zhang

**Affiliations:** ^1^Teaching and Learning Group, The Royal New Zealand Police College, Wellington, New Zealand; ^2^College of Foreign Languages, Huazhong Agricultural University, Wuhan, China; ^3^Faculty of Education and Social Work, The University of Auckland, Auckland, New Zealand; ^4^College of Foreign Languages, Shanxi University of Finance and Economics, Taiyuan, China

**Keywords:** personalized mobile learning, m-learning, self-regulated learning (SRL), English-as-a-foreign language (EFL), grammar learning

## Abstract

This study developed a personalized mobile-assisted system with a self-regulated learning (SRL) mechanism to facilitate English-as-a-foreign-language (EFL) students’ learning of grammar. A quasi-experimental design, involving an experimental group (*n* = 278) and a control group (*n* = 320), was adopted to examine its effectiveness on students’ grammar learning. The experimental group, who had full access to the functions of the system (i.e., recommendations, feedback and an e-portfolio package), used the system to learn grammar, complete grammar exercise, and engage in for a semester, while the control group only use the system to submit weekly assignments. The results suggest that the experimental group obtained significantly higher scores in English grammar tests than the control group and the system benefited both genders with no significant differences. The findings provide empirical evidence in support of the effectiveness of this system in improving students’ grammar test scores, indicating its value as a supplementary tool to conventional classroom teaching of English grammar *via* supporting learners’ SRL development in a m-learning context. The pedagogical implications for applying this system in classrooms and beyond were discussed.

## Introduction

For English-as-a-foreign-language (EFL) learners, the significance of the explicit teaching of English grammar has seldomly been questioned (Aarts et al., 2012). In recent times, with the rapid development of wirelessly networked technologies, mobile devices have been increasingly employed in EFL instruction, including the teaching of English language grammar. Besides pushing the boundaries of time and place limitations, mobile learning provides learners with a personalized learning experience regarding their abilities, interests and preferences (Abu-Al-Aish and Love, 2013; Davison and Argyriou, 2016; Montiel et al., 2020). It affords students the choice of what, where, when and how they would like to learn (Crompton, 2013; Broadbent et al., 2020). In light of these advantages, educators of EFL learning have been incorporating mobile devices into their instruction and various mobile learning systems were thus developed to support students’ EFL learning in their spare time, including the learning of vocabulary (e.g., Chen et al., 2019) and the development of reading (e.g., Chang and Hsu, 2011; Wang, 2016) and communicative speaking skills (e.g., Loewen et al., 2020). However, meta-analytic reviews of language learning studies using mobile devices showed that grammar instruction in EFL learning received little attention (Duman et al., 2015; Chen et al., 2020).

Furthermore, as most learning activities with mobile devices are designed to be carried out by students independently in the absence of support and guidance from instructors, m-learning could be a challenge for them as they need to determine for themselves when and how to engage with which information and content and persist in learning despite difficulties (Kizilcec et al., 2017; Broadbent et al., 2020). This could be especially true in the learning of English grammar as it is viewed as a daunting task for many EFL learners (e.g., Tuan and Doan, 2010; Zhou, 2019). Effective mobile learning necessitates not only behavioral and cognitive engaging in learning whenever and wherever necessary, but also the willingness to monitor, control, and regulate the learning process (Sha et al., 2012). Researchers thus argued that a prerequisite for effective mobile learning is learners’ self-regulated learning (SRL) abilities (e.g., Sha et al., 2012; Chen et al., 2019). Learners need to possess the abilities to metacognitively, motivationally, and behaviorally engage in their learning processes to attain learning goals in m-learning contexts (Sha et al., 2012; Seifert and Har-Paz, 2020). Previous studies have shown that students’ SRL abilities could critically influence their language learning performance in m-learning contexts, such as English vocabulary (e.g., Chen et al., 2019), English literature (e.g., Seifert and Har-Paz, 2020), and EFL reading comprehension (e.g., Chang et al., 2011; Chen et al., 2014). Despite the critical role SRL plays in language learning with mobile devices, few studies were conducted to facilitate EFL students’ learning of English grammar *via* promoting their SRL abilities in mobile learning contexts. To address these research gaps, a personalized mobile-assisted system with a self-regulated learning mechanism was developed in this study to help EFL students in the learning of English grammar. Moreover, as previous studies indicated the possibility that the two genders might have different performance after using technology-based learning tools (e.g., Chen and Huang, 2014), this study also examined whether this personalized mobile-assisted system with a self-regulated learning mechanism benefited the two genders differently regarding their learning of English grammar.

## Literature Review

### Utilizing Mobile Devices in English Grammar Instruction

One of the current trends in second/foreign language teaching and learning is the increasing reliance on mobile technology both in and beyond the classroom (Sung et al., 2016; Reinders and Benson, 2017; Loewen et al., 2020). Mobile devices, such as laptops, personal digital assistants, and mobile phones, provide potentially beneficial affordances for language learning, including flexibility in time and location of study, accessibility of information, and adaptability to personal study habits (e.g., Petersen and Sachs, 2016; Reinders and Pegrum, 2016; Loewen et al., 2020). In the field of EFL/ESL learning, various language learning applications (e.g., ABA English, Busuu, Duolingo, Rosetta Stone, and Voxy), spanning both mobile devices and personal computers, have thus been developed to help second/foreign language learners to access learning material anytime and anywhere (Loewen et al., 2020).

Technological advances have also changed the way L2 grammar is taught and learnt and various technology-based pedagogies for L2 grammar were developed and applied (see Heift and Vyatkina, 2017, for a summary). Nevertheless, meta-analyses of language learning studies using mobile devices showed that among the language skills in EFL learning, vocabulary learning and the development of listening and speaking skills received the greatest attention, while studies on grammar instruction were lacking (Duman et al., 2015; Chen et al., 2020).

Despite the limited number of studies in this line, results of previous studies have shown promise in employing mobile devices as a supplement to classroom teaching to facilitate students in learning English grammar. For instance, Li and Hegelheimer (2013) designed a mobile application, *Grammar Clinic*, to help ESL students at the tertiary level to improve the grammar accuracy of their writing. This mobile learning application involved students identifying and correcting sentence-level grammatical errors (i.e., sentence fragments, run-on sentences, ambiguous expressions, etc.). The findings showed that participants performed significantly better in the post- grammar test than the pre-test. The number of grammar errors in their writing also reduced significantly after using *Grammar Clinic*. Similarly, Chu et al. (2017) also leveraged the mobile learning devices to teach English grammar and obtained positive results. In their study, a mobile gaming system was developed to help EFL elementary school students to learn English grammar in a structured way. A grammar concept mapping strategy was taught *via* the mobile system and the instructional effects were examined *via* a quasi-experimental design. The experimental group significantly outperformed the control group in the English grammar test, demonstrating the effectiveness of the mobile gaming system in helping students to internalize grammar structures. These previous results served as evidence of English grammar teaching and learning in mobile-learning contexts.

### Self-Regulated Learning in Mobile Learning Contexts

Besides the shortage of research into grammar instruction for EFL/ESL learners in mobile learning contexts, another impetus for this study is the lack of grammar learning mobile applications that incorporated self-regulated learning (SRL). As a construct, SRL refers to the degree to which learners actively participate in their learning process; it involves “cognitive, affective, motivational, and behavioral components that provide the individual with the capacity to adjust his or her actions and goals to achieve desired results in light of changing environment conditions” (Dörnyei, 2005, p. 191). SRL is regarded as a dynamic process whereby “students activate and sustain cognitions that are systematically oriented toward an attainment of their goals” (Schunk and Zimmerman, 1994, p. 309). In the self-regulating process, learners undertake task analysis, goal setting, and plan formation to help them achieve learning goals; they apply strategies to facilitate task completion and engage in monitoring and regulation of motivation and engagement (Zimmerman, 2000, 2011). Namely, for learning to be effective, students need to intentionally activate, sustain, and adjust their cognition, affect, and behavior to achieve their learning goals (Zimmerman and Schunk, 2011). In this sense, for online learning to be successful, learners also need to possess self-regulatory learning skills.

In fact, SRL could play an especially critical role in mobile learning contexts compared with other types of learning activities because of its distinguishing characteristic of autonomy (e.g., You and Kang, 2014; Zhao et al., 2014; Lin et al., 2017; Viberg and Andersson, 2019). Mobile learning is distinguished by ubiquity, which refers to not only learning anytime and anywhere, but also “widespread,” “just-in-time,” and “when-needed” (Peng et al., 2019, p. 175). Specifically, learners are required to assume the responsibilities of determining what, when, where, and how to learn, as well as to behaviorally and cognitively engage in learning whenever and wherever it is needed. Researchers thus have posited that the ubiquity of mobile learning intrinsically calls for SRL abilities (e.g., Sha et al., 2012). In mobile learning contexts which are highly learner-centered and self-regulated, students’ SRL skills are viewed as a precursor to effective learning (e.g., Sha et al., 2012; Broadbent and Poon, 2015; Littlejohn et al., 2016; Viberg and Andersson, 2019). In the field of second/foreign language learning, empirical studies have shown that students’ SRL abilities were positively associated with their use of the technology to plan, monitor, and evaluate their language learning process, thereby influencing their performance (e.g., Lai and Gu, 2011; Lai, 2013).

Furthermore, previous studies demonstrated that mobile learning might be more effective than traditional teaching in cultivating students’ SRL abilities as it provided a means by which students can exercise agency to control their behavior and cognition (e.g., Joo et al., 2000; Sha et al., 2012; Olga and Andersson, 2019; Chen and Hsu, 2020). Mobile-assisted learning systems with an SRL mechanism were thus developed to facilitate EFL/ESL students’ learning *via* improving their SRL abilities (e.g., Shih et al., 2005; Chen, 2009; Kondo et al., 2012; Chen and Huang, 2014; Chen et al., 2019). For instance, Kondo et al. (2012) developed a mobile-assisted language learning module that involved an SRL learning cycle. Results showed that this module greatly facilitated students’ EFL learning and helped them to obtain higher scores on listening and reading tests than the pre-tests. Similarly, Chen et al. (2019) designed an English vocabulary mobile application with an SRL mechanism which greatly improved students’ learning performance and motivation for vocabulary learning. In their study, two classes of Grade five students were randomly assigned into an experimental condition, where students used the vocabulary learning application with an SRL mechanism, or a control condition, where students used a vocabulary learning application without the SRL mechanism. The findings showed that the experimental group significantly outperformed the control group in learning performance and motivation for vocabulary learning, providing evidence in support of the critical role SRL played in facilitating language learning.

Despite the prominent role SRL plays in EFL/ESL learning in mobile learning contexts, there were few systems with SRL mechanisms for the learning of English grammar. How to effectively improve EFL/ESL students’ learning of English grammar *via* promoting their SRL abilities in mobile learning contexts remains largely unexplored.

### Personalized Mobile Learning

Besides creating an environment for students to engage in the SRL, mobile learning can provide a personalized learning experience to promote language learning (Traxler, 2011; Crompton, 2013; Xie et al., 2019). Compared with traditional, teacher-centered classrooms where students are likely to receive the same education inputs at the same pace, mobile learning can create a more personalized learning environment *via* providing learning activities depending on their characteristics, such as their performance, preferences, profiles, status, and requirements (Kinshuk et al., 2009; Peng et al., 2019). A characteristic of many existing mobile learning systems that were effective in helping EFL/ESL students is personalized learning (e.g., Chen and Chung, 2008; Chen and Li, 2010; Hsu et al., 2013; Bringula et al., 2017). These systems used functions such as recommendation, portfolio, and feedback to achieve personalization in mobile learning contexts. For instance, in the mobile learning system developed by Chen and his colleagues (e.g., Chen et al., 2006; Chen and Chung, 2008; Chen and Hsu, 2008; Chen and Li, 2010), a recommendation mechanism was included to facilitate the development of EFL students’ reading abilities. The system would recommend vocabularies and reading materials to learners based on their abilities. Results showed that the personalized mobile system with a recommendation function significantly improved EFL learners’ reading performance and learning interests and enabled them to outperform those in the control condition.

Other researchers designed systems with an e-portfolio function, which could record students’ learning preferences, history and replies, to provide a personalized learning environment (e.g., Tzouveli et al., 2008; Wu et al., 2014; Beckers et al., 2016). For example, Wu et al. (2011, 2014) included an e-portfolio in their system to guide EFL learners to read and students demonstrated great gains in their English reading performance. In their system, learners’ portfolio recorded information such as learners’ reading abilities and the level of difficulty of the reading material they preferred, which contributed to the accuracy of the guidance provided in their system.

Moreover, the feedback function has also been found to contribute to improvements in students’ language learning performance. Palomo-Duarte et al.’s (2016) designed a mobile learning system that provided real-time task performance feedback to students. Once a student completed a task, the system offered him/her feedback on whether it was performed correctly or incorrectly. The authors reported that their system greatly facilitated the students’ foreign language learning.

Despite the fact that personalized mobile learning systems have exerted positive effects on EFL learning, few systems were developed to offer a personalized learning context for grammar learning. To fill the research gaps mentioned above, the present study was conducted to develop a personalized mobile-assisted system with an SRL mechanism to help EFL students’ learning of grammar.

### Influences of Gender Differences on the Effectiveness of Mobile Learning

Mixed results were reported regarding the effectiveness of m-learning for the two genders in existing literature and a primary reason lay in their different attitudes toward m-learning. An early study of secondary school students by Young (2000) showed that male learners were more confident in using learning technologies than female learners. Similarly, Cai et al.’s (2017) meta-analysis study also indicated that male learners tended to possess more favorable attitudes toward technology use than female learners. Another line of research, however, found no significant differences between the two genders in their attitudes toward technology (e.g., Bain and Rice, 2006).

In the specific domain of EFL language learning, inconsistent findings were also reported regarding the effects of mobile learning on different genders’ language learning performance. For instance, Chen and Huang (2014) research with EFL learners demonstrated that the two genders developed differently in their English reading performance after they used a digital reading system with SRL learning mechanism. These findings, however, were not in line with Chen et al.’s (2019) study, which indicated that an English vocabulary app with SRL learning mechanism benefited EFL learners in English vocabular learning, regardless of their genders.

In the field of grammar learning, few studies were performed to explore the effectiveness of mobile learning for the two genders. The current study therefore also examines the effectiveness of the personalized mobile-assisted system, developed to facilitate EFL learners’ learning of English grammar *via* promoting their SRL abilities, on different genders.

## The Current Study

The present study adopted a quasi-experimental research design to evaluate the effectiveness of our system on students’ performance in English grammar tests. As well as to determine whether the system works effectively, this study also explored the effectiveness of the system for the two genders. The following research questions guided this study:

1. Did the system help the experimental group to obtain higher scores in the English grammar test than the control group?
2. If yes, did the two genders in the experimental group progress differently in the English grammar tests?

### Research Context

This research was conducted in a senior high school in mainland China, where all students were enrolled in English courses (240–300 min) per week with explicit instructions of curriculum (Ministry of Education of the People’s Republic of China, 2000). The aims of the English courses were to improve students’ grammatical knowledge, lexical knowledge, reading ability, and test-taking skills. Furthermore, these students’ English proficiency were closely linked to a large-scale high-stakes test—the College Entrance Examination. Consequently, students’ lexico-grammatical knowledge and the ability of taking tests are key advantages for their future success. Therefore, this study aims to explore the grammatical development of high school students in such a context. More information about the research is described below.

### Participants

A total of 598 EFL learners were recruited from a senior secondary school using the convenience sampling technique. They were randomly assigned into the experimental group (*n* = 278) or the control group (*n* = 320). All participants were informed of their right to withdraw from the project at any time of the programme. No statistical differences were found between the two groups concerning their average age, gender proportion, length of English learning (see Table 1). At the time of study, none of the participants had used any grammar learning mobile applications with an SRL mechanism before.

**TABLE 1:** Participant demographic information.

Group	*n*	Male%	Year level	Average length of English learning (*SD*)
Experimental group	278	56.1%	10	6.27 (0.46)
Control group	320	62.2%	10	6.14 (0.78)

### Instruments

#### The Personalized Mobile-Assisted System With an Self-Regulated Learning Mechanism

**FIGURE 1:**
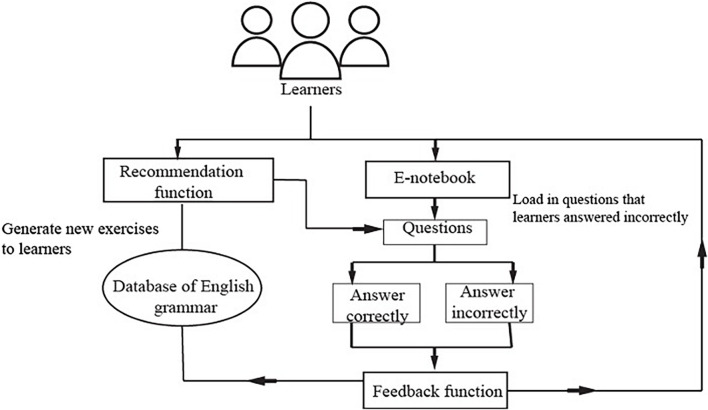
The operating mechanism of the system. Modified from Chen et al. (2019) operating mechanism of a quiz module.

The teaching content included in the system was designed under the guidance of *Senior High School English Curriculum*, a nationally sanctioned syllabus that is used as the benchmark for curriculum development for high school students in China. Specifically, this system aims to teach students grammar rules including basic parts of speech (i.e., nouns, pronouns, verbs, adjective, adverbs, prepositions, conjunctions, articles) and how to use them in sentences, and other basic English grammar rules such as tense (i.e., simple tense, progressive and perfect tense), subjunctive mood, adverbial clause, objective clause, direct/indirect statement, attributive clause. The operating mechanism, system components, and functions are described below. Figure 1 presents an overview of the operating mechanism of our system.

##### The operating mechanism of the system

**FIGURE 2:**
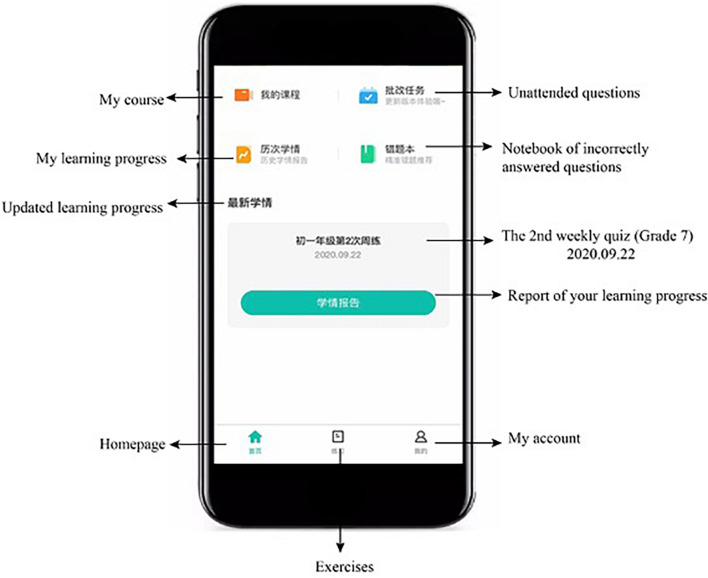
User interface of setting goals and monitoring progress.

**FIGURE 3:**
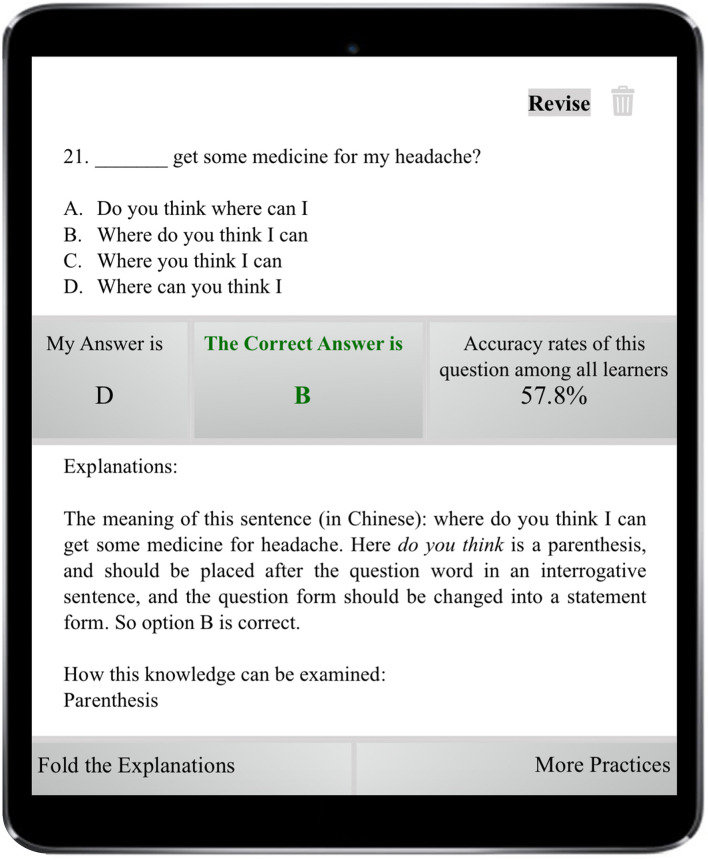
User interface of a summarized report of students’ answers.

**FIGURE 4:**
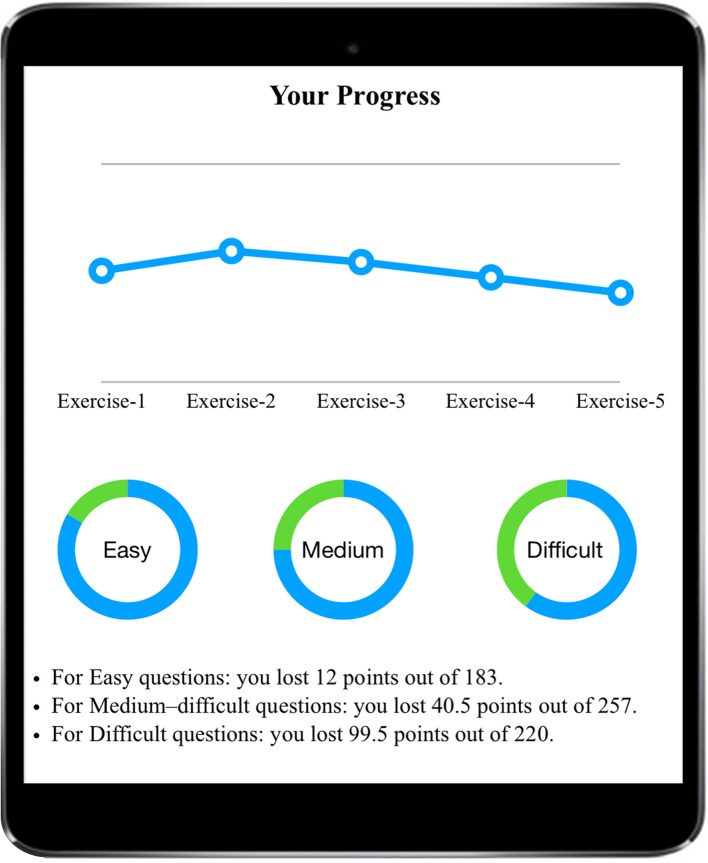
User interface of monitoring progress.

**FIGURE 5:**
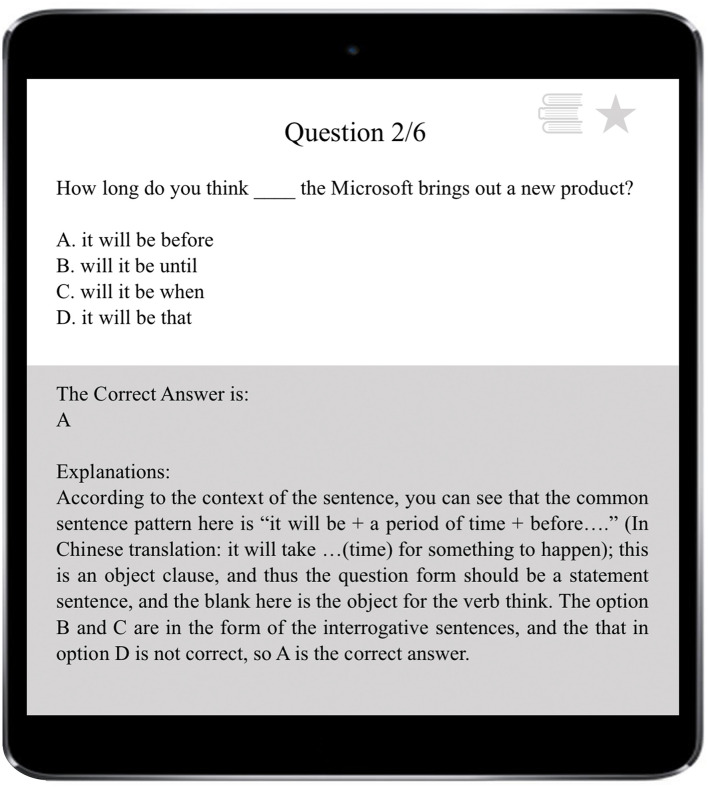
User interface of detailed explanations.

This system was developed to support students’ learning of English grammar *via* encouraging them to intentionally activate, adjust, and sustain their cognition and behavior to achieve learning goals. In light of existing SRL literature which depicts SRL as an active and constructive process (e.g., Zimmerman, 2002; Zimmerman and Schunk, 2011), this system involves students setting goals for themselves, reflecting upon their learning progress, evaluating their performance, and making adjustments for further learning in the grammar learning process. To promote students to engage in the SRL process, the system was designed to provide some degree of freedom for students to set learning goals, selecting tasks, monitoring and their learning progress and evaluating their learning activities (Sha et al., 2012). Once the learner logins into the system using the account name and password, s/he sets the learning goal by choosing either to review the previously, wrongly answered questions or continue with where s/he left last time (i.e., unattended questions), as shown in Figure 2. If it is the first time s/he logins into the system, s/he sets the learning goal *via* deciding on the year level of the learning material and the grammar rules to be learnt. The system then assigns various sets of exercises to the learners depending on their learning goals with each set consisting of six questions. Upon completion of a set of exercises, learners receive a summary of their performance, including the number of correctly answered questions and system-generated comments. All incorrectly answered questions are highlighted and displayed in this report (see Figure 3). Learners then are provided with two options: to receive a detailed explanation of these incorrectly answered questions or move on to learn new materials, as displayed in Figure 4. This selection requires learners to reflect on their learning progress and to evaluate their previous performance. For those who would like to receive help with the wrongly-answered questions, the system provides explicit explanations of the grammar rules these questions are concerned as well as other examples to demonstrate this rule (Figure 5). For those who choose to move on to learning new grammatical rules, the system offers another set of exercises and learners engage in the learning process described above.

##### Functions of the system

This system creates a personalized learning experience for learners with three main functions, recommendations, feedback and an e-portfolio, to facilitate their SRL of English grammar. The recommendation function recommends exercises to learners based on their learning goals and previous performance. These recommended exercises were selected from a large question pool, in which each question has been categorized regarding its difficulty level, the grammatical rule it contains, and the frequency of its occurrence in previous examinations. For instance, the English multiple-choice question “*Unless extra money ____, the theatre will close. A. was found, B. finds, C. is found, D. found*” is of medium-level difficulty (i.e., it scores 3 out of a 5-point scale with 5 representing very difficult) and it examines learners’ understanding of adverbial clauses and the passive voice of verbs. Its occurrence in previous examinations was not frequent (i.e., it scores 2 out of a 5-point scale with 5 representing very frequent). The system uses these indexes to identify the current level of learners’ abilities in using the grammatical rules, to understand learners’ weaknesses *via* their answers to questions, and to recommend exercises from the database of questions depending on learners’ performance.

**FIGURE 6:**
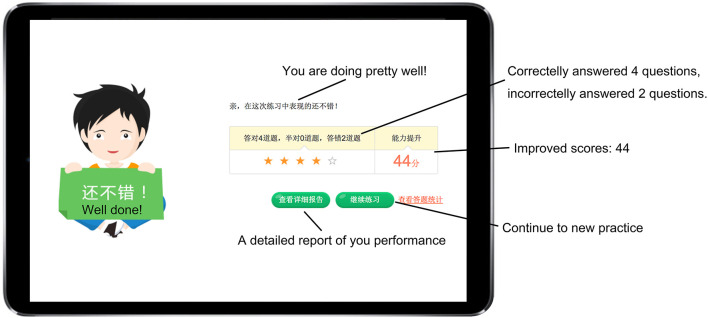
User interface of the progress recoding.

Feedback function provides question-based feedback, including the correct answer, explicit explanations, information about how the relevant knowledge has been examined in past examinations, and the correct rate of each question upon completion of the exercises. The system records students’ progress *via* tracing the changes in students’ performance and providing a monthly report summarizing their previous incorrectly answered questions. The system also offers information on the difficulty level of these questions and the knowledge that they might not have mastered yet, as shown in Figure 6.

**FIGURE 7:**
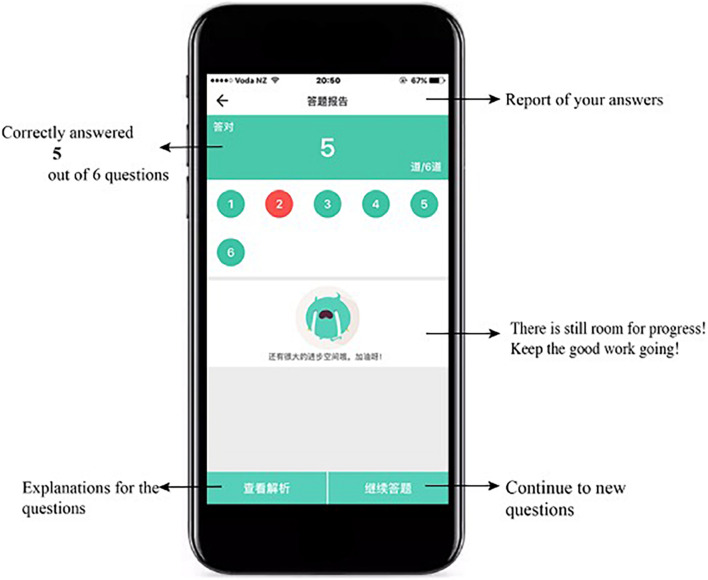
User interface of the e-notebook.

Lastly, the system sets up an e-portfolio through which learners can track their learning history and monitor their learning performance. In the e-portfolio, there is an e-notebook that collects all the questions that were answered incorrectly. Figure 7 shows an example of the e-notebook. Learners can re-answer these questions and/or practice similar questions recommended by the system.

#### The Pre- and Posttests of English Grammar

Each of the pre- and posttests was a cumulative review of the grammatical structures that were taught in class as well as in the system. As described in the previous section The “Personalized Mobile-Assisted System With an Self-Regulated Learning Mechanism,” the teaching content of grammatical structures in the system was designed in line with the requirement of the nationally sanctioned syllabus for high school students in China. The grammar tests focused exclusively on the grammar points and consisted of various discrete items to assess participants’ knowledge of the target grammatical structures, which were in line with previous studies in this line that also utilized such a type of tests to measure whether there were improvements in students’ grammar knowledge after mobile learning (e.g., Turner, 2017; Loewen et al., 2020). Both the pre- and post- grammar tests comprised of 50 multiple choices questions, designed by two experienced EFL teachers, to examine the grammar structures that were introduced in the system, with each question worth two points. Multiple choice tests do not measure linguistic production and therefore can be appropriately used to measure students’ knowledge of grammar within the framework of grammar consciousness-raising (Nutta, 2013). Students were required to select a grammatically correct answer from four choices. The internal consistency of the tests was acceptable for both the pre- and post-tests (above.80). A sample of the questions on the pre- and posttests is provided in Appendix A.

### Procedure

At the beginning of the semester, participants in both groups were invited to complete the pre-grammar test in classroom environments. Students in the experimental group were then invited to participate in a training session in the following week to familiarize themselves with the functions and processes of using the system. After that, the 16-week treatment started and the participants from the experimental group used the system to study English grammar for at least 15 min every day. There was no time limitation for using the m-learning system and students were encouraged to use the system when needed. They could choose which lessons to study in the system and which functions to use. Also, they were invited to complete a set of weekly exercises, which were assigned into their accounts every Monday morning with a notification sending to their mobile devices. They would also receive a detailed report summarizing their performance of the previous week on a weekly basis. Participants in the control group, however, had limited access to the functions of the system. They could only login the system to complete grammar exercises as assignments and submit their answers. Differing from the exercises provided for the experimental group, which were selected and recommended based on students’ learning history and performance, the exercises used in the control group were chosen based on the year level without considering their learning progress. To measure the instructional effects of the system on students’ performance in grammar texts, participants in both groups completed the post- grammar test after the treatment.

### Data Analysis

To examine whether the two groups developed differently in their English grammar tests scores over time, two-way mixed ANOVA was used with conditions (experimental and control) and time (pre-and post-test) as independent variables and English grammar test scores as the dependent variable. *Post hoc* Tuckey’s tests were conducted to perform pairwise comparisons to determine at which time point between-group differences emerged. Similarly, two-way mixed ANOVA was applied to examine whether the two genders in the experimental condition developed differently in their tests scores over time with time and gender (male and female) as independent variables and their test scores as the dependent variable. Partial eta-squared (η*_*p*_^2^*) were adopted to estimate effect sizes for ANOVA and Cohen (2013) d for paired-samples *t*-test. Assumptions for the mixed-ANOVA, including normality, homogeneity of variance, and linearity, were checked prior to the analyses.

## Results

To answer the two research questions, the findings concerning the comparison of the two groups’ performance in the grammar tests are presented first, followed by the results of comparing the performance of the two genders from the experimental group.

**FIGURE 8:**
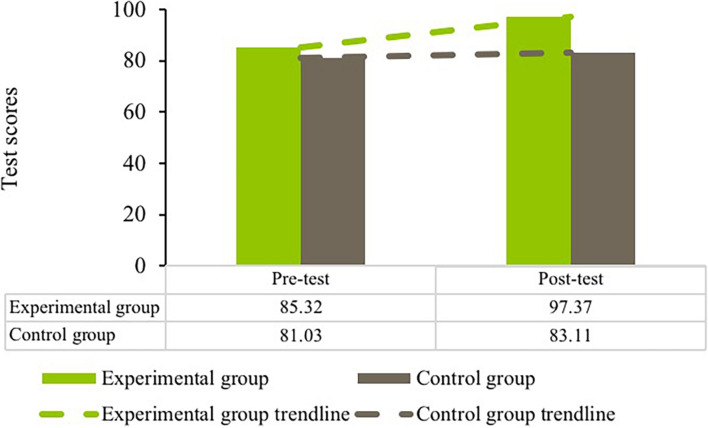
Participants’ test scores in pre- and post-tests.

It can be seen from Figure 8 that the experimental group scored higher than the control group in the post-test, while the two groups obtained similar scores in the pre-test. A significant interaction between time and condition was detected in the English grammar test scores [*F*(1, 1192) = 12.42, *p* < 0.001, partial η*^2^* = 0.01]. Tuckey’s pairwise comparisons indicated that the experimental group significantly outscored the control group in both the pre- and post-tests (all *p* = 0.00). Therefore, ANCOVA was performed on the post-test scores with their pre-test scores being controlled as covariates and a significant result returned [*F*(1, 595) = 91.33, *p* = 0.00, partial η*^2^* = 0.13]. These results suggested that the experimental group outperformed the control group in the English grammar test scores after the intervention.

Table 2 presents the descriptive information on English grammar test scores for each gender in pre- and post-tests. Two-way mixed ANOVA did not find a significant interaction between gender and time in the test scores [*F*(1, 552) = 0.73, *p* = 0.39, partial η*^2^* = 0.01]. The main effect of time was significant [*F*(1, 552) = 9.24, *p* = 0.00, partial η*^2^* = 0.02]. Tuckey’s tests showed that the differences lay between different time points for each gender (both *p* = 0.00). These results indicated that both genders progressed similarly in their English test scores.

**TABLE 2:** Descriptive information on test scores for each gender in the experimental group.

	Male (*n* = 156)		Female (*n* = 122)	
		
	*M*	*SD*	*M*	*SD*
Pre-test	86.04	27.16	84.39	21.76
Post-test	97.65	27.41	97.01	22.76

## Discussion

### Positive Influences of the Personalized Mobile-Assisted System on Learners’ Grammar Test Scores

The results showed that the participants who used the personalized mobile-assisted system significantly outperformed the control group in the grammar test scores, indicating greater improvements of the treatment group in the concrete grammar focus of the course than the comparison group. The findings could serve as initial evidence in support of the effectiveness of the personalized m-learning system with SRL an mechanism in facilitating EFL students’ learning in the target grammatical structures. Namely, this study yields empirical support for the feasibility of employing mobile devices in helping students to learn the target grammatical structures as supplementary to classroom instruction in an EFL context. The results obtained in the current study, which was among the first few to investigate EFL grammar instruction with middle school students using mobile devices, also echo with empirical evidence in support of the positive effects of m-learning systems with an SRL mechanism on students’ language learning performance in EFL settings (e.g., Kondo et al., 2012; Chen and Huang, 2014; Chen et al., 2019) and extend its application in the area of grammar instruction. Researchers (e.g., Krashen, 2014; Loewen et al., 2019) argue that mobile learning is good for developing explicit, decontextualized linguistic knowledge such as grammatical structures, which can be accessed through reflection. In the current study, the participants were encouraged to evaluate their current performance, set goals for their learning based on their understanding of their current level, and reflect on the areas that they had not mastered yet and track their learning progress while they were using the system, which supported their learning of the discrete, explicit linguistic knowledge. Researchers proposed that when learning technologies were deliberately designed to support self-regulation, students’ academic performance would improve (e.g., Kitsantas et al., 2015). Also, the positive results of this study also collaborate with those of previous m-learning systems that created a personalized learning environment for learners. To facilitate students’ learning of the grammatical structures as well as their SRL abilities, the system developed in the present study involved three main features: feedback, recommendation, and e-portfolio, to provide personalized guidance to each student depending on their prior performance, learning needs, and learning progress through the learning of grammar. Our results indicated that these features, each of them to be effective on its own, to be effective when combined together in teaching grammar in m-learning contexts.

### No Gender-Related Influences on the Improvements in Tests Scores

No significant differences were found between the male and female students in their grammar test performance after using this system, indicating that the two genders benefited similarly from the system in learning English grammar. These findings appear to suggest that the an SRL mechanism implemented in our system were effective across both genders on grammar improvement, as measure by their grammar test scores. The results are congruent with those of previous studies in which SRL mechanisms were also adopted in m-learning systems such as Chen et al. (2019) study. In Chen et al.’s (2019) investigation, they designed an English vocabulary learning system with SRL mechanisms and found that both genders exhibited significantly greater performance in the vocabulary test. The authors concluded that the SRL mechanism, promoted with their system, seemed to be equally effective for the two genders in helping them to learn English vocabulary.

Our findings, however, were different from other studies such as Chen and Huang (2014) which reported that their web-based reading system with an SRL mechanism was more effective in promoting the reading comprehension for the female participants than the male counterparts. They argued that a gender difference in reading comprehension with their system existed and the two genders might use different SRL strategies during their learning process in the m-learning environment. The existing literature is inconclusive regarding whether there were gender differences relating to SRL in m-learning contexts. Some studies reported that gender differences existed in motivational aspects of SRL and behavioral learning strategy in m-learning contexts (e.g., Lee, 2002), while other studies such as Yukselturk and Bulut (2009) research found no significant differences between the genders regarding SRL learning and learning performance in m-learning context.

A possibility that could account for the inconsistent results reported in the previous studies as well as the current one is that a technological gender gap, which used to influence the performance of the two genders, has been narrowing down greatly with the widespread use of mobile technology for both genders (Cai et al., 2017; Reychav and McHaney, 2017). Although prior studies described computing and technology use as a masculine activity and the female possessed relatively negative attitudes toward it and were not as active as the male in technology-related activities, recent studies showed that female learners hold strong beliefs that they had a similar level of competence to that of male learners in using the Internet and expressed positive attitudes toward m-learning (Li and Kirkup, 2007; Al-Emran et al., 2016; Cai et al., 2017; He and Zhu, 2017). The findings obtained in the current study implied that the female learners were as skillful as the male counterparts in using our m-learning system, as the two genders developed similarly in their grammar test scores. It is possible that in some of the early studies where this technological gender gap still played an important role, the learning performance of the two genders developed differently.

### Limitations

Certain limitations need to be considered when interpreting the findings of this study. First, participants’ prior knowledge of SRL was not examined. Although the participants were assigned into the conditions randomly, whether the two groups were equivalent in the use of SRL strategies had not been examined. Their prior perceptions of SRL motivational beliefs as well as their use of SRL strategies could also contribute to the changes in their academic performance (Cueli et al., 2017). We acknowledge that this limitation could have weakened our findings and recommend further work to address this limitation.

Also, the grammar exercises used in the current study were limited to the form of multiple-choice questions, which might have not fully captured the nuanced changes in students’ grammar knowledge. Multiple-choice questions were used in the system to allow for relatively efficient analysis of students’ answers and easy identification of the knowledge they had not mastered. Different types of questions should be used in future studies to provide more reliable information on students’ learning outcomes.

## Conclusion, Implications, and Future Directions

In an attempt to address the research gap that EFL grammar learning with mobile devices has not received much attention, the present study designed a personalized mobile-assisted system with an SRL mechanism and examined its effectiveness with EFL learners in grammar learning. Our findings demonstrate the positive influences of this system on students’ grammar test performance, regardless of their genders, indicating its value as a supplementary tool to traditional classroom instruction of English grammar. The results obtained in this study also add to a growing body of literature on mobile learning in support of its effectiveness for EFL learning as well as the effects of promoting SRL in m-learning contexts on EFL learners’ performance. Despite certain limitations of this study, it can be concluded that the personalized m-learning system was effective for both genders in improving their English grammar test scores.

The present study yields several pedagogical implications. Firstly, the system can be applied as a supplement to conventional classroom teaching and learning of English grammar *via* supporting the development of learners’ SRL beyond classrooms. This system provides a personalized and flexible learning environment for learners to use in their leisure time, thus allowing them to study grammar at their own pace and plan, monitor, and control their learning progress through the recommendation, feedback and e-portfolio functions without the assistance of teachers. The personalized instruction provided in the system, together with the SRL mechanism, could provide learners with individualized learning experiences that facilitate their autonomy and independence as well as promote their performance outside the classrooms. Moreover, the system proposed in this study can recommend appropriate English grammar exercises to individual learners depending on their learning goals, current level, and prior performance and facilitate their grammar learning. Hence, it can also be integrated with formal classroom activities and applied as a useful tool for teachers to help students achieve specific learning goals.

Besides addressing the limitations of the current study, future studies are needed to explore several issues. First, other functions that were shown to be effective in previous m-learning systems (e.g., Shih et al., 2010; Wu and Huang, 2017), such as game functions or scaffolding functions could be developed for our proposed system to increase students’ motivation to learn grammar. For instance, further studies could compare the instructional effects of the system with and without a game-based function on students’ grammar performance, SRL strategy employment, or motivation for learning English grammar. It is also important for future studies to consider students’ learning characteristics, such as their cognitive learning styles or self-regulated learning types, and to examine whether students of various learning profiles would benefit differently from this system. Researchers (e.g., Chen et al., 2016, 2019) reported that students of different cognitive styles exhibited varying learning performance in technology-supported learning environments. Whether students of various cognitive styles would progress similarly in their grammar performance after using this system could be the focus of further research.

## Data Availability Statement

The raw data supporting the conclusion of this article will be made available by the authors, without undue reservation.

## Ethics Statement

Ethical review and approval was not required for the study on human participants in accordance with the local legislation and institutional requirements. Written informed consent to participate in this study was provided by the participants’ legal guardian/next of kin.

## Author Contributions

XW conceived and designed the research project, collected the data, and contributed to the writing and editing of literature review, research methodology, and discussion. JC performed the data analysis, produced figures and tables, and contributed to the writing and editing of research methodology, results and discussion. TZ designed the research project, contributed to the design of research instruments, and the writing and editing of research methodology. All authors contributed to the article and approved the submitted version.

## Conflict of Interest

The authors declare that the research was conducted in the absence of any commercial or financial relationships that could be construed as a potential conflict of interest.

## Publisher’s Note

All claims expressed in this article are solely those of the authors and do not necessarily represent those of their affiliated organizations, or those of the publisher, the editors and the reviewers. Any product that may be evaluated in this article, or claim that may be made by its manufacturer, is not guaranteed or endorsed by the publisher.
