# Supplementary material for: Facilitating English Grammar Learning by a Personalized Mobile-Assisted System With a Self-Regulated Learning Mechanism
Source: Front Psychol. 2021 Oct 7;12:624430. doi: 10.3389/fpsyg.2021.624430 (PMC8529239; doi:10.3389/fpsyg.2021.624430)
Supplement: Supplementary file 1 [file Data_Sheet_1.docx]

**Appendix A**: A sample of the items on the pre- and posttests

**Pretest**

Directions: *For each of the questions there are four choices marked A), B), C) and D). You should decide on the best choice to complete each sentence.*

1. I can't remember _____________ made the teacher give Mary the permission to leave the class earlier.
A. that it was what B. what it was that C. what was it that D. that was it what

2. He let out an ______________ cry, "we've won!"
A. excited B. exciting C. excite D. excites

3. -Is football John's favourite sport?
 -Yes. __________ football, baseball is his greatest love.
A. Near to B. Except C. Beside D. Next to

4. Do you think regular exercise ___________ good health?
A. benefit from B. reach for C. make for D. go for

5. Have you applied ___________ Mr Black _____________ the post? It's worth a try.
A. for; to B. with; for C. with; about D. to; for

6. _______ the loud noise going on in the workshop, I can hardly __________ on my lessons.
A. As; put B. As; concentrate C. With; rely D. With; concentrate

7. _________, I've never seen anyone who's as capable as Kate.
A. As long as I have travelled B. Now that I have travelled so much

C. As I have travelled much D. Much as I have travelled

8. The big fire lasted as long as 24 hours ______________ it was brought under control.
A. after B. before C. since D. while

9. The discovery of new evidence led to ______________.
A. the thief having been caught B. the thief to be caught

C. catch the thief D. the thief being caught

10. -Would you have helped her had it been possible?
 -Yes, but I __________ busy with my work.
A. was B. had been C. have been D. am

**Posttest**

Directions: *For each of the questions there are four choices marked A), B), C) and D). You should decide on the best choice to complete each sentence.*

1. ___________ on the small island, the tower has been seriously damaged by the sea water?
A. Being lain B. Lain C. Being located D. Located

2. In this workshop, the output of July was three times _____________ of January.
A. that B. this C. one D. it

3. -Well, where did you spend your night that day?
　 -At __________.
A. where it is called Grand Hotel B. what is called Grand Hotel

C. which is called Grand Hotel D. that is called Grand Hotel

4. The thief was brought in, his hands ____________ behind his back.
A. tying B. tied C. being tied D. having tied

5. The journey around the world took the old sailor nine months, __________ the sailing time was 226 days.
A. of which B. during which C. from which D. for which

6. She stood on a brick to make herself ___________ a bit taller.
A. looking B. looked C. to look D. look

7. Did your speech ______________ the audience?
A. get through to B. get across to C. get through D. get across

8. Du's unique voice ___________ especially to young people.
A. attracts B. draws C. catches D. appeals

9. The old woman stood still with her eyes ____________ the picture.
A. fixing in B. fixing on C. fixed in D. fixed upon

10. I feel it is you who ___________ for the accident.
A. is to blame B. is to be blamed C. are to blame D. are to be blamed
